# Andrographolide and 4-Phenylbutyric Acid Administration Increase the Expression of Antimicrobial Peptides Beta-Defensin-1 and Cathelicidin and Reduce Mortality in Murine Sepsis

**DOI:** 10.3390/antibiotics11111629

**Published:** 2022-11-15

**Authors:** Albert Bolatchiev, Vladimir Baturin, Elizaveta Bolatchieva

**Affiliations:** 1 Laboratory of Antimicrobial Peptides (Former Microbiota Laboratory), Department of Clinical Pharmacology, Stavropol State Medical University, 355000 Stavropol, Russia; 2 Department of Clinical Pharmacology, Stavropol State Medical University, 355000 Stavropol, Russia; 3 AlboGene, LLC, 355000 Stavropol, Russia; 4 Department of Anatomy, Stavropol State Medical University, 355000 Stavropol, Russia

**Keywords:** antimicrobial peptide, antibiotic resistance, andrographolide, 4-phenylbutyric acid, defensin, cathelicidin

## Abstract

Antibiotic resistance is a global threat and requires the search for new treatment strategies. Natural antimicrobial peptides (AMPs) have pronounced antibacterial, antiviral, antifungal, and antitumor activity. AMPs’ clinical use is complicated by the high synthesis costs and rapid proteolytic degradation. The search for small molecules, inducers of endogenous AMP expression, could become a new approach. Here, we investigated for the first time the effect of seven small molecules (andrographolide, levofloxacin, azithromycin, montelukast, 4-phenylbutyric acid, rosuvastatin and valsartan) on AMP (beta-defensin-1, hBD-1 and cathelicidin, LL-37) serum levels in rats. In control groups, the level of hBD-1 was 295.0 (292.9–315.4) pg/mL, and for LL-37, it was 223.8 (213.3–233.6) pg/mL. Andrographolide (ANDR) and 4-phenylbutyric acid (4-PHBA) administration significantly enhanced the level of both AMPs. The hBD-1 level was 581.5 (476.3–607.7) pg/mL for ANDR and 436.9 (399.0–531.6) pg/mL for 4-PHBA. The LL-37 level was 415.4 (376.2–453.8) pg/mL for ANDR and 398.9 (355.7–410.1) pg/mL for 4-PHBA. Moreover, we have shown that these compounds reduce mortality in a murine model of sepsis caused by a carbapenem-resistant *Klebsiella aerogenes* isolate. From our point of view, these small molecules are promising candidates for further study as potent AMP inducers. The data obtained allow the development of new strategies to combat antibiotic resistance and infectious diseases.

## 1. Introduction

The global threat of antimicrobial resistance (AMR) is one of the greatest challenges for modern medicine [1]. It is estimated that by the middle of the 21st century, more than 10 million deaths annually may be associated with AMR [2]. Recent work has shown that more than 1.2 million deaths were directly attributable to AMR, and 4.95 million deaths were associated with bacterial AMR globally in 2019 [3]. Such a rapid spread of AMR is forcing the search for new strategies to fight resistant infections.

One of the promising methods may be the development of new drug candidates based on antimicrobial peptides (AMPs) [4]. AMPs are an essential component of the innate immune system and provide anti-infective protection against bacteria, viruses, protozoa, parasites, and fungi [5]. Among natural mammalian antimicrobial peptides, alpha- and beta-defensins, as well as cathelicidins, are of great interest [6]. The mechanism of AMPs antibacterial action is based on a direct effect on the cell wall by membrane permeabilization [7].

Several strategies of AMPs’ clinical application can be distinguished: (1) the use of native AMPs of natural origin; (2) the design of new and unique AMPs and/or modification of natural AMPs; (3) the design of genetic constructs (for example, mRNA or viral vectors) expressing target AMP.

For example, in previous studies, we have shown that AMPs of natural origin, human beta-defensin-3 and epinecdin-1 by *Epinephelus coioides,* can increase survival by up to 83% in mice infected with a lethal dose of carbapenem-resistant strains of *Klebsiella pneumoniae* and *Pseudomonas aeruginosa* [8]. This effect was observed even with a single intraperitoneal injection of 100 μg of AMP. In the same study, we showed that AMPs can become an effective tool to overcome carbapenem resistance.

Various de novo design methods for unique and patentable AMPs have also been previously demonstrated. For these purposes, different rational design methods are suitable, including modern bioinformatic techniques and artificial intelligence [9,10,11]. In our other recent work, we experimentally verified for the first time that a recurrent neural network can be used to generate new peptides with pronounced antibacterial activity against carbapenem-resistant isolates of Gram-negative bacteria, as well as reducing mortality in experimental sepsis in mice [12].

Biotechnological approaches demonstrate that the target AMP can be expressed efficiently [13,14,15]. This approach is probably the most appropriate for the development of anticancer peptides [16].

All these strategies are promising; however, we believe that another one can be applied—these are small molecules, inducers of the expression of endogenous AMPs [17]. This approach can become an effective and cheaper alternative to the exogenous administration of AMP. However, there are currently only limited studies on eukaryotic cell cultures to search for small molecules that can induce the expression of endogenous AMPs [17].

In this study, we for the first time evaluated the effect of seven small molecules (andrographolide, levofloxacin, azithromycin, montelukast, 4-phenylbutyric acid, rosuvastatin and valsartan) on their ability to change the levels of endogenous AMPs (beta-defensin-1 and cathelicidin) in rats. Those candidates were chosen based on the results of in vitro studies [17] or empirically.

## 2. Results

**Figure 1:**
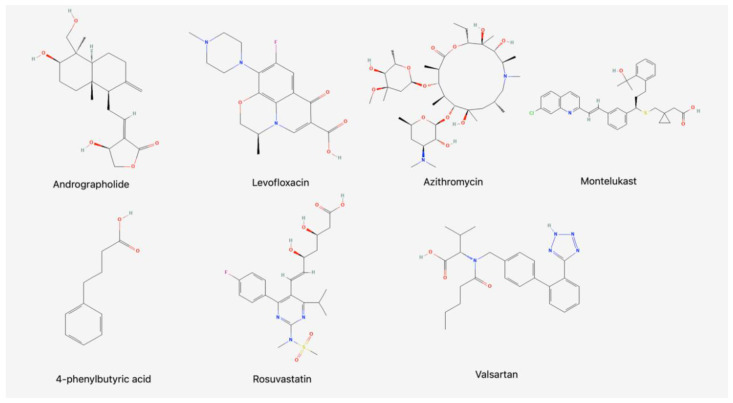
Two-dimensional structures of small molecules investigated in this work.

Seven different compounds (Figure 1) were administered once a day for three consecutive days, and on the fourth day, blood was collected to evaluate the serum levels of beta-defensin-1 and cathelicidin in rats.

### 2.1. Andrographolide, Levofloxacin, 4-Phenylbutyric Acid, Rosuvastatin and Valsartan Increase the Serum Level of Beta-Defensin-1

In the control group, the level of beta-defensin-1 was 295.0 (292.9–315.4) pg/mL. Azithromycin and montelukast had no effect on endogenous beta-defensin-1 serum levels with 290.3 (278.8–304.1) pg/mL (*p* = 0.3442) and 355.3 (329.8–374.5) pg/mL (*p* = 0.1304), respectively.

Levofloxacin administration demonstrated increased levels of beta-defensin-1—439.4 (374.5–485.8) pg/mL (*p* = 0.006993), as well as rosuvastatin—354.0 (340.4–368.8) pg/mL (*p* = 0.02813) and valsartan—400.5 (374.2–429.4) pg/mL (*p* = 0.002953).

**Figure 2:**
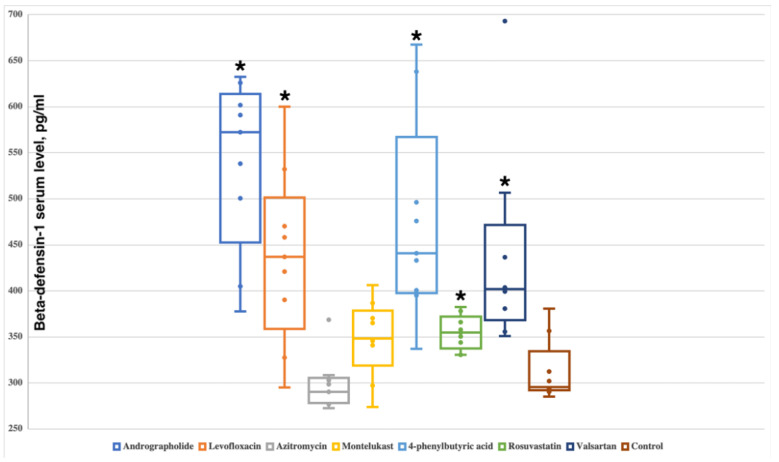
The effect of administration of the studied small molecules on the level of beta-defensin-1 in rats. Boxplots show median, lower and upper quartiles, minimum and maximum, and individual data points. Significant differences from the control group are marked with an asterisk.

The highest effect on the expression of beta-defensin-1 in rats was shown when used andrographolide—581.5 (476.3–607.7) pg/mL (*p* = 0.0003108) and 4-phenylbutyric acid—436.9 (399.0–531.6) pg/mL (*p* = 0.0006216) (Figure 2 and Supplementary file S1).

### 2.2. Andrographolide, Levofloxacin, Azithromycin, Montelukast and 4-Phenylbutyric Acid Increase the Serum Level of Cathelicidin

In the control group, the level of endogenous cathelicidin was 223.8 (213.3–233.6) pg/mL. It was shown that rosuvastatin and valsartan do not affect the level of cathelicidin—serum levels were 239.4 (223.1–261.1) pg/mL (*p* = 0.3282) and 238.6 (179.7–272.2) pg/mL (*p* = 0.7984), respectively.

**Figure 3:**
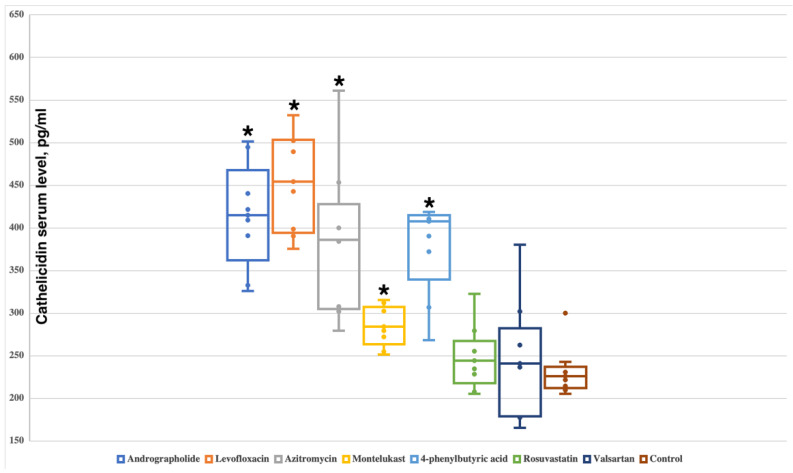
The effect of administration of the studied small molecules on the level of cathelicidin in rats. Boxplots show median, lower and upper quartiles, minimum and maximum, and individual data points. Significant differences from the control group are marked with an asterisk.

All other investigated compounds had significant differences from the control group with the following cathelicidin serum levels: andrographolide—415.4 (376.2–453.8) pg/mL (*p* = 0.0001554); levofloxacin—465.8 (396.4–502.6) pg/mL (*p* = 0.0001554); azithromycin—392.0 (306.2–414.7) pg/mL (*p* = 0.0003108); montelukast—282.3 (267.7–304.7) pg/mL (*p* = 0.002953); 4-phenylbutyric acid 398.9 (355.7–410.1) pg/mL (*p* = 0.0003108) (Figure 3).

### 2.3. Andrographolide and 4-Phenylbutyric Acid Increase Survival in Murine Model of Sepsis

Since andrographolide and 4-phenylbutyric acid increased AMP expression in vivo, we hypothesized that the administration of these compounds prior to infection may affect sepsis mortality. We injected these small molecules (andrographolide, 80 mg/kg, and 4-phenylbutyric acid, 500 mg/kg) intraperitoneally for three consecutive days in mice. On the fourth day, the mice were infected with a lethal dose of a carbapenem-resistant *Klebsiella aerogenes* strain. Survival was accessed for 5 days by Kaplan–Meier.

**Figure 4:**
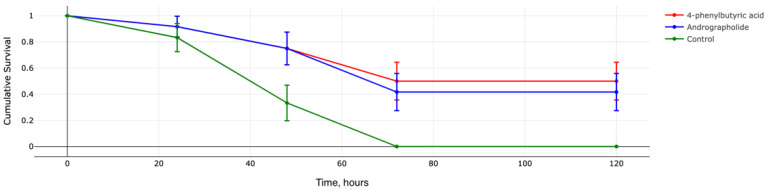
Survival function with standard error. Survival was assessed by the Kaplan–Meier method. Statistically significant differences with *p* = 0.0394525 were found between the control group (sterile saline) and the two experimental groups (andrographolide–80 mg/kg and 4-phenylbutyric acid–500 mg/kg).

In the control group, all animals died on the third day after infection (0% survival). Both andrographolide and 4-phenylbutyric acid increased survival with *p* = 0.0394525 compared to control. In total, 41.6% of animals administered with andrographolide survived 5 days after infection. In the 4-phenylbutyric acid group, survival was 50% (Figure 4 and Supplementary file S1).

## 3. Discussion

In this work, we have shown for the first time in vivo that a few small molecules can enhance the production of endogenous AMPs, such as beta-defensin-1 and cathelicidin. We demonstrated that the administration of andrographolide, levofloxacin or 4-phenylbutyric acid can increase the level of both investigated AMPs. Rosuvastatin and valsartan are able to stimulate the expression of beta-defensin-1. Azithromycin and montelukast might activate the cathelicidin production.

Of course, a serious limitation of our work is that we do not know which mechanism underlies the discovered phenomenon of the induction of endogenous AMPs expression. However, the data obtained allow us to ask a number of questions for further in-depth experiments to explain the mechanism of action.

Andrographolide is the main active component of the herb *Andrographis paniculate* and has direct wide antibacterial and anti-inflammatory activity [18]. In lung epithelial cells, it was shown that andrographolide can upregulate the expression of human beta-defensin-2 in a dose-dependent manner through the p38 mitogen-activated protein kinase and nuclear factor kappa B pathways [19]. Sechet E. et al. have identified the epidermal growth factor receptor as the target of andrographolide, which increased the phosphorylation of histone H3 on serine S10 and the recruitment of the c-Fos, c-Jun, and Elk1 or c-Myc transcription factors at the human beta-defensin-3 promoter, inducing its overexpression [20]. Interestingly, in the same work, the authors studied cathelicidin, the level of which did not change after andrographolide exposition.

To date, there is only one study that assessed the level of AMP in the use of fluoroquinolones and macrolides. Baturin et al. showed that the beta-defensin-1 levels were increased by 45% after josamycin therapy and by 80% after levofloxacin use in women with pelvic inflammatory disease [21]. These antibiotics have completely different chemical structures and points of application. Thus, we cannot assume the molecular mechanism of the effect of increased AMP expression observed by us after the administration of levofloxacin and azithromycin.

Previously Steinmann J. et al. demonstrated that 4-phenylbutyric acid upregulate the expression of beta-defensin-1 and cathelicidin in epithelial cells and in macrophages [22]. This effect was based on the increased expression of genes encoding target peptides. It should also be noted that Ottosson and colleagues have discovered a few potent new AMP-inducers, aroylated phenylenediamines [23], which have some structural similarities with 4-phenylbutyric acid.

In general, most of the studies carried out on cell cultures show that the mitogen-activated protein kinases, nuclear factor kappa B and histone deacetylase signaling pathways play vital roles in the induction of AMP expression [17]. However, given that all the molecules we studied have a completely different chemical structure, we believe that further studies are necessary to investigate the mechanism of action in vivo, as well as the search for new small-molecule inducers of endogenous AMPs.

For screening validation of our data, we tested the effect of two small molecules, andrographolide and 4-phenylbutyric acid, in an experimental model of sepsis. We have previously shown that even small amounts of exogenous AMPs can greatly reduce mortality in vivo [8,12]. We considered that in order to achieve the effect of enhancing the expression of endogenous AMPs, it is necessary to inject andrographolide and 4-phenylbutyric acid for three consecutive days. We then infected the animals with a lethal dose of a carbapenem-resistant *Klebsiella* isolate. Survival analyses have shown that these small molecules can decrease mortality in vivo. That is, for the first time, we can put forward a hypothesis that the administration of small molecules—inducers of endogenous AMP expression—can have a positive effect on experimental infection.

## 4. Materials and Methods

Animal studies were approved by the Ethics Committee of Stavropol State Medical University (protocol No. 95 of 18 February 2021) and were performed on male Wistar rats with an average weight of 180–200 g (7–8 weeks old) and female ICR (CD-1) mice with an average weight of 30 g (5–6 weeks old). In vivo studies were carried out in accordance with the Code of Ethics of the World Medical Association (Declaration of Helsinki, EU Directive 2010/63/EU for animal experiments) and reported in compliance with the ARRIVE guidelines [24].

### 4.1. Study of the Influence of Small Molecules on the Level of Endogenous AMPs

The animals were grown and kept in the vivarium of Stavropol State Medical University, 4 rats in each cage with free access to food and water, under a 12 h light/dark cycle and 65% humidity. The animals were randomly divided into eight groups (*n* = 8 rats for each group): (1) andrographolide 80 mg/kg intraperitoneally (i.p.); (2) levofloxacin 200 mg/kg i.p.; (3) azithromycin 30 mg/kg i.p.; (4) montelukast 10 mg/kg orally; (5) 4-phenylbutyric acid 500 mg/kg i.p.; (6) rosuvastatin 25 mg/kg orally; (7) valsartan 10 mg/kg orally; (8) control group—sterile saline i.p. (Figure 1).

Andrographolide (purity ≥98%) and 4-phenylbutyric acid (purity ≥98.5%) were purchased form Sigma-Aldrich (USA); other compounds were purchased from a pharmacy chain: levofloxacin (Sanofi, France), azithromycin (Kraspharma, Russia), montelukast (Sandoz, Slovenia), rosuvastatin (AstraZeneca, UK), valsartan (Novartis, Switzerland).

All drugs were administered once a day for three consecutive days. On the fourth day of the experiment, the blood was collected from the tail vein for enzyme-linked immunosorbent assay (ELISA). The *Rattus norvegicus* beta-defensin-1 and cathelicidin blood levels were measured using ELISA according to the manufacturer protocols on a commercial basis in the immunology laboratory of the Center for Clinical Pharmacology and Pharmacotherapy (Stavropol, Russia). Both ELISA kits were purchased from Cloud Clone corp., USA. In this study, euthanasia was not required before the planned end of the experiment. At the end of the experiment, rats were euthanized with ketamine (360 mg/kg) + xylazine (40 mg/kg) i.p. administration followed by decapitation.

### 4.2. Effect of Andrographolide and 4-Phenylbutyric Acid on Survival in Experimental Sepsis

To assess the impact of the studied small molecules on survival, a murine model of lethal generalized infection was used, previously described by us [12]. The mice (4 animals in each cage) were housed in temperature-controlled rooms at 24 °C and 65% humidity with a 12 h/12 h light/dark cycle and water and food availability ad libitum. The mice were injected with bacterial suspension of the carbapenem-resistant *K. aerogenes* isolated in 2022 from intensive care unit patient of Stavropol State Regional Clinical Hospital.

Identification and antibiotic resistance of *K. aerogenes* were determined using the disk diffusion method as part of a routine microbiological study in accordance with the European Committee on Antimicrobial Susceptibility Testing protocols in the Department of Clinical Microbiology of the Center of Clinical Pharmacology and Pharmacotherapy [25].

*K. aerogenes* suspension was prepared from a fresh morning culture in approximate concentration of 4.5 × 10^9^ CFU/mL (McFarland turbidity standard of 15). DEN-1 densitometer (Biosan, Latvia) was used to determine the turbidity of the suspension. *K. aerogenes* suspension were dissolved in sterile saline and injected intraperitoneally (the total volume of injected fluid did not exceed 250 μL per animal). There were 3 groups, *n* = 10 in each group. The first group, control, received sterile saline; the second group–andrographolide; the third group—4-phenylbutyric acid. For three consecutive days, the second and third groups received andrographolide 80 mg/kg and 4-phenylbutyric acid 500 mg/kg, respectively, once a day, i.p. On the fourth day, all three groups were infected with 150 μL of *K. aerogenes* suspension, pprox.. 6.75 × 10^8^ CFU per mice. Survival was assessed every 24 h for 5 days. Moribund animals were euthanized to avoid unnecessary distress.

### 4.3. Statistical Analysis

To compare the level of serum AMPs between groups we used two sample Mann–Whitney U test. The results are presented as median and in brackets—Q1 and Q3 quartiles. Two-tailed *p*-values were calculated for each group in comparison with control.

We used Kaplan–Meier online calculator (https://www.statskingdom.com/kaplan-meier.html, accessed on 26 October 2022) and StatPlus v8.0.3 for macOS (AnalystSoft Inc., Walnut, CA, USA) for statistical analysis of survival.

## 5. Conclusions

In this work, we demonstrated for the first time in vivo that the administration of andrographolide, levofloxacin, 4-phenylbutyric acid, rosuvastatin, valsartan and azithromycin can enhance the production of endogenous AMPs. Further studies are needed to understand the molecular mechanism of the data obtained. Additionally, it has been demonstrated that andrographolide and 4-phenylbutyric acid might increase survival in a murine model of sepsis. The results obtained can be used to develop new strategies for combating infectious diseases.

## Supplementary Materials

The following supporting information can be downloaded at: https://www.mdpi.com/article/10.3390/antibiotics11111629/s1, File S1: Raw data. The raw data show the ELISA measurements and survival analysis.
